# Improving Vibrational Spectroscopy Prospects in Frontline Clinical Diagnosis: Fourier Transform Infrared on Buccal Mucosa Cancer

**DOI:** 10.1021/acs.analchem.2c02496

**Published:** 2022-09-26

**Authors:** Edward Duckworth, Arti Hole, Atul Deshmukh, Pankaj Chaturvedi, Murali Krishna Chilakapati, Benjamin Mora, Debdulal Roy

**Affiliations:** † Swansea University, Singleton Park, Swansea, SA28PP Wales, United Kingdom; ‡ Advanced Centre for Treatment, Research and Education in Cancer, Kharghar, Navi Mumbai 410210, India; § Tata Memorial Center, Head and Neck Surgical Oncology, Dr. E Borges Road, Parel, Mumbai 400012, India; ∥ Center for Interdisciplinary Research, D. Y. Patil Dental College, Nerul, Navi Mumbai 400706, India; ⊥ Department of Life Sciences, Homi Bhaba National Institute, Mumbai 400094, India

## Abstract

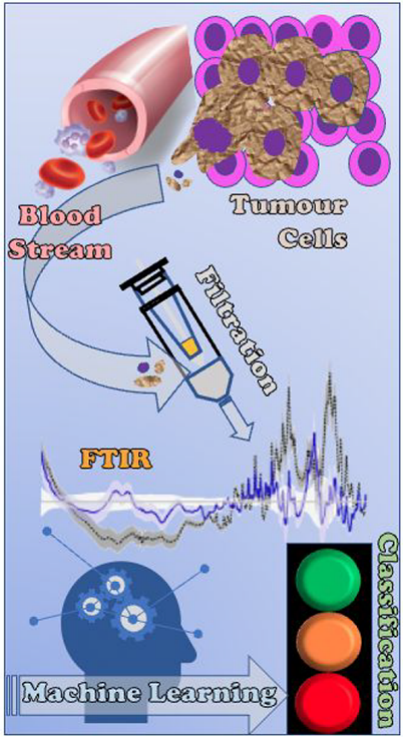

We report a novel method with higher than 90% accuracy in diagnosing buccal mucosa cancer. We use Fourier transform infrared spectroscopic analysis of human serum by suppressing confounding high molecular weight signals, thus relatively enhancing the biomarkers’ signals. A narrower range molecular weight window of the serum was also investigated that yielded even higher accuracy on diagnosis. The most accurate results were produced in the serum’s 10–30 kDa molecular weight region to distinguish between the two hardest to discern classes, i.e., premalignant and cancer patients. This work promises an avenue for earlier diagnosis with high accuracy as well as greater insight into the molecular origins of these signals by identifying a key molecular weight region to focus on.

## Introduction

Vibrational spectroscopy as a method to discern between cancerous and healthy patients has been a popular field of study in recent years.^1,2^ The potential for this field of research is great, especially when the focus is on spectral analysis of biofluids, which can allow for minimally invasive detection of these diseases through simple swabs or blood/urine tests. This in turn could allow for more readily available screening for these diseases, leading to earlier detection. However, due to the lack of transferability of the results to the clinical setting, little of this positive impact has occurred.^1,2^

The ability to detect cancers at an early stage has a dramatic effect on the cost of treating the disease. For example, early stage colon cancer treatment costs can increase nearly 4-fold when having to treat at a late stage.^3^ In practice, the effectiveness of screening has been demonstrated: a UK study found 46 cases of cancer from computed tomography scanning 2500 people, with 80% of the cases being early stage.^4,5^ Biofluid spectroscopy could provide a fast, easy, affordable, minimally invasive method for cancer screening by comparing an unknown patient sample with a premade database of known healthy and known cancerous samples to determine if there is an affliction.^1,6,7^

Blood is a particularly useful biofluid for inspection due to its high protein and lipid concentration. In addition, low concentration nucleic acid fragments and changes in these levels are some of the best indicators of disease. Much of the current research is focused on subsets of blood: the plasma and serum.^1,2^ In whole blood, hemoglobin and other red blood cell associated molecules can interfere with the spectra. Therefore, the plasma is preferred as the molecular concentrations within are more sensitive to a disease. Serum is a subset of plasma without the coagulating factors, which enables easier storage and use. Without these natural coagulants present, other decoagulating chemicals do not need to be added. Characterizing a serum sample, by quantifying the minute quantity of markers within, is key for being able to tell if it is diseased or not.^8^

One of the best methods for this analysis is vibrational spectroscopy, particularly for it being a nondestructive procedure, allowing us to examine a sample in as close to a natural composition as possible without labeling. Much of the investigations are proof-of-principle studies.^9−13^ These typically demonstrate the potential of Fourier transform infrared (FTIR) or Raman spectroscopy to distinguish between diseased and healthy samples in a relatively small sample set. Of these studies, many of them are investigating cancers in the attempt to distinguish characteristic spectral biomarkers for them.

Recently, there has been a study demonstrating better quantification of molecules, such as glycine in serum, by only using the <10 kDa fraction as it removes large obscuring signals from globulin (>80 kDa) and albumin (>60 kDa).^14^ Potentially even subsets of serum will be more accurate for the identification of spectral biomarkers for certain diseases. Further research using attenuated total reflectance (ATR)-FTIR has been done in this area, looking at using ultrafiltration on samples to get better detection of the low molecular weights.^15,16^ Therefore, it is necessary to see if this effect can be transferred to transmission FTIR.

Subsequently, Roy et al. looked at if the same 10 kDa cutoff could be useful for detecting hepatitis in human serum with ATR-FTIR.^17^ The results demonstrated a significantly lower accuracy from the <10 kDa subset. It can be hypothesized that a viral infection operates differently from a cancer and should therefore produce different signatures in the blood. The choice of <10 kDa also remains unjustified as the potentially obscuring molecules mentioned are >60 kDa. It would also be useful to see if this cutoff is optimal. We chose to test these hypotheses in this study.

It is apparent that by finding biomarkers for cancer within a subset of the serum, the ability to discern the molecules providing the signal would be improved without the unnecessary obscuring molecules and their intense signals. Thereby, the spectral biomarker can be connected to the real change in blood molecular concentration caused by the cancer, or the body’s reaction to it. Finding this connection would be a major step in the field of spectral diagnosis.

Buccal mucosa was chosen as a suitable cancer to test the potential of fractionating serum before analysis due to recent Raman spectroscopy-based research into its potential for screening by Sahu et al.^18^ The feasibility of classification was explored before being followed up by a larger and more comprehensive study.^19^ The latter study contained suitable premalignant and related disease controls and produced sensitivity and specificity values of 64 and 80% respectively in determining the presence of an abnormality. Higher values were obtained for determining the correct abnormality from the glioma, premalignant, and oral cancer options used in the model. It was noted that these values are comparable to current screening techniques.

In this study, serum samples were filtered and segmented into different molecular weight windows to see if, by removing obscuring molecules, detection accuracy can be improved for buccal mucosa cancer.

To surmise, the key hypotheses being tested are the following:

- Is transmission FTIR effective at diagnosing buccal mucosa?
- Are the key signaling molecules for this cancer being obscured by larger molecules in the blood?
- What molecular weight region is best to investigate?

## Experimental Method

### Patient Selection

A premalignant control was selected as this would best emulate a practical diagnosis scenario where the disease of interest should be discernible from similar, nonmalignant diseases. A study on ovarian cancer performed similar control, effectively discerning cancer patients from other benign ovarian patients.^20^ A healthy control is used as a reference and to potentially allow quantification of the cancer severity if patient outcomes are monitored.

Here 126 patients were analyzed, with 42 of them being cancer patients, 40 premalignant, and 44 healthy. A full summary of these is given in Table S4, with additional detail in Table S1. In this study, certain factors could influence the serum spectra such as age, sex, diet, lifestyle habits, e.g., smoking, pre-existing conditions, or other diseases. Aside from diet, efforts were made to eliminate or control these, and the method of choosing a narrow molecular weight window was aimed at minimizing them. The available samples for buccal mucosa patients were predominantly male. Therefore, only male patients were selected for this initial study. All premalignant and cancer patents were tobacco users, and a healthy tobacco user control was used. The patient’s reference diagnoses were clinically determined in TATA hospital.

### Sample Preparation

The blood serum was collected, under ethical guidelines and approved by the ethical committees in India, from the Advanced Centre for Treatment, Research and Education in Cancer (ACTREC) and D.Y. Patil University Navi Mumbai, India. Written informed consent was obtained from all the subjects as well. Samples were stored at −80 °C until being thawed for analysis. The serum was segmented into two fractions using Millipore 500 μL 50 kDa centrifugal filters. The centrifuge was run for 20 min at 14000*g*. Whole serum, <50 kDa low molecular weight (LMW) and >50 kDa high molecular weight (HMW) fractions were analyzed (see Figure S1). For the FTIR measurement, each fraction was diluted in a 1:24 ratio of sample to Milli-Q ultrapure water before 500 μL was deposited on a 25 mm diameter CaF_2_ slide purchased from Crystran, ensuring the surface was covered to the edges, and left to dry overnight for analysis. Dilution was to ensure absorption was in the correct range for the FTIR measurement and to reduce the variable deposition “coffee ring effect”^1^ (Figure S2 and Figure S3).

Henceforth, the molecular windowing experiment will refer to the FTIR measurements conducted on a narrower molecular weight range of the serum. This is achieved by filtering twice using upper and lower cut off filters. For the molecular windowing experiment, serum samples were first filtered through 100 kDa filters. Both filtrate and concentrate were collected, and the filtrate was moved on to further filtering using 50, 30, 10, and 3 kDa filters until 6 subsets of serum were produced. Each of the fractions, i.e., 0–3, 3–10, 10, 30, 30–50, 50–100, >100 kDa and whole serum were all analyzed for comparison (Figure S4).

### Spectral Acquisition

FTIR spectra were acquired with a PerkinElmer “Spectrum Two” FTIR spectrometer used in transmission mode. The resolution was 4 cm^–1^, and spectra were acquired for 5 s with 10 accumulations over a range of 750–4000 cm^–1^.

### Preprocessing of Spectra

Spectra were trimmed to the 1000 data points in the 800–1800 cm^–1^ fingerprint region of most interest, then preprocessed with a background correction using the Asymmetric Least Squares Smoothing (ALSS) baselining algorithm.^21^ This was followed by average normalization.

### Postprocessing of Spectra

Instead of directly feeding 1000 dimensions of a spectrum to a support-vector machine model (SVM), the spectra were first analyzed to extract up to 50 principal components (PCs) (orthogonal features), which were subsequently analyzed using a support-vector-machine model (see Figure S5 and Figure S6). This dimensionality reduction should help reduce the chances of a model overfitting. Linear PCA-SVM and complete “leave-one-out” cross validation were chosen due to effectiveness from earlier studies and in internal testing.

The model was benchmarked against the community standard on Data Optimisation Model Evaluation^22^ (DOME) methodology (see Table S3).

Further investigation was carried out to compare the cross-validated accuracies obtained from SVM and LDA alone, i.e., using the FTIR spectra directly, as opposed to using the relevant number of PCs for PCA-SVM analysis (see Table 1). It can be clearly observed that the PCA-SVM analysis produced higher accuracies on average compared to SVM or LDA analysis alone. Table S7 offers additional information on these classifications.

**Table 1:** FTIR Cross-Validation Sensitivity (Sen), Specificity (Spc), and Principal Components (PCs) Results for Classifying between Buccal Mucosa Cancer Samples from Healthy and Premalignant Using PCA-SVM^a^

	Classification of cancer and healthy			Classification of cancer and Premalignant			Classification of cancer and all other			Average cross-validation accuracies (%)		
Fraction	Sen (%)	Spc (%)	PCs	Sen (%)	Spc (%)	PCs	Sen (%)	Spc (%)	PCs	PCA-SVM	LDA	SVM
LMW	88	88	29	83	84	46	65	81	30	82.3	76.5	77.2
HMW	94	82	10	83	83	15	81	89	24	86.1	83.9	83.1
Whole	89	86	29	90	84	27	84	90	43	87	82.7	79.7

a Post-cross-validation results using LDA or SVM alone are also included for comparison, demonstrating a similar accuracy trend but with lower accuracies overall.

## Results and Discussion

### Comparison of Whole Sera with LMW and HMW Fractions

**Figure 1:**
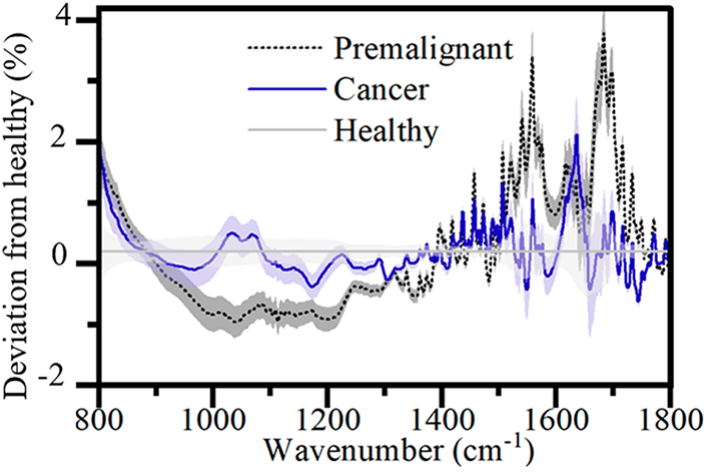
Difference in the average spectra of cancer and premalignant patient serum from healthy for whole serum. Error in faded color around each line shows level of distinction for each spectrum.

The average spectral differences for whole serum can be seen in Figure 1.

**Figure 2:**
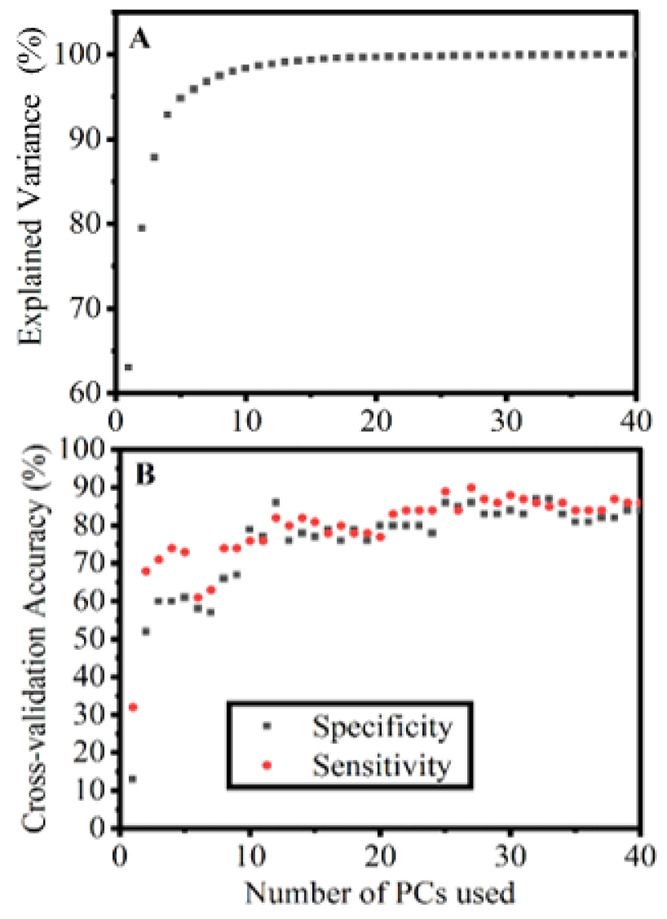
(A) Explained variance graph depending on the number of principle components (PCs) used. (B) Cross-validated sensitivity and specificity values dependent on the number of PCs used in the model. Example graph to demonstrate how the accuracy plateaus after a sufficient number of principle components. The cross-validated accuracy does not decrease after a point as the SVM algorithm ignores the unnecessary components and minimal or no overfitting occurs. The two graphs mimic one another; the plateau in panel B starts at 25 principle components whereas in panel A there is 99.84% variance explained. This example is from the classification of the whole cancer vs premalignant subset.

The background subtracted spectra (preprocessed) were used to calculate the PCs. An example of the variation of accuracy and specificity with the number of PCs is shown in Figure 2. The highest accuracy and specificity combinations were chosen for analysis of spectra from low molecular weight (<50 kDa), high molecular weight segments (>50 kDa), and the whole sera. The cross-validated sensitivity and specificity results for classifying the spectra are summarized in Table 1. The separability of the groups is high all round with >80% accuracy. There is a 95% confidence interval of approximately 4% for classifications on the cohort size used.

The FTIR results for whole serum demonstrates the ability to effectively distinguish between healthy, premalignant, and cancerous serum samples with high (>85%) accuracy. Additionally, the ability to classify using the spectra from low and high molecular weight subsets of the serum was demonstrated, although no obvious benefit was evident. Therefore, we zoomed-in to narrower molecular windows to investigate further.

### Narrower Molecular Windowing

**Figure 3:**
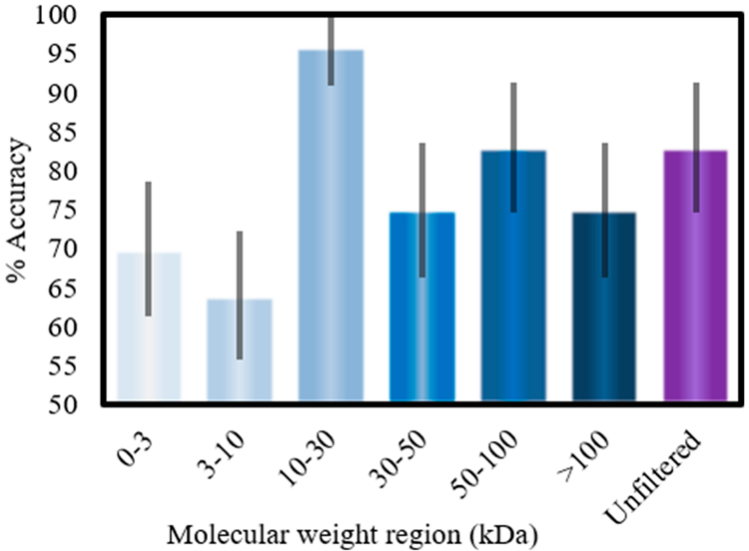
Classification accuracies between FTIR spectra of premalignant and cancer patients for different serum molecular weight subsets (molecular windows). The 95% confidence interval is shown by the gray lines over the bars. The 10–30 kDa window performed significantly better than the whole serum.

We continued to search for the narrow molecular weight windows of the blood serum where the accuracy is the highest. In this experiment (shown in Figure 3), the 10–30 kDa subset performed significantly better than the whole serum, producing a highly accurate cross-validated classification where all the patients were classified correctly. Even though the sample size for this experiment is small, the confidence interval depicted in Figure 3 is higher. However, the results indicate a valuable 10–30 kDa window of interest for further investigation.

It is worth noting that although our hypothesis that the key signaling molecules were being obscured by the larger proteins in the serum^12^ was not disproved, it did not result in a higher classification accuracy. In this regard, the results were similar to the hepatitis study by Roy et al.^15,17^ However, our spectra are majorly different after the reduction of the contribution of albumin, globulin, and other high weight components.

It is valuable to discern the root biological cause of the spectral shifts observed. Knowing what molecular weight fraction the key information is present in as well as the key peaks of interest can be used together to help identify potential biomarker molecules.

The identification of the 10–30 kDa region as providing the best overall classification accuracy (above 90%) indicates that the molecular weight splitting method can potentially have significant value, especially if this specific region can be examined in further studies.

## Conclusions

The potential of FTIR for screening of buccal mucosa cancer is demonstrated, with classification accuracy of 87% for the whole serum. The additional use of ultrafiltration provided more information about the signal’s origins, with contributing factors present in both the high and low molecular weight regions. Furthermore, the molecular windowing showed even greater promise from its even higher classification accuracy for the 10–30 kDa window. This can inform a follow-up study into the root cause of the spectral biomarker identified. Other benefits of a narrower molecular window include suppressing external factors, such as alteration of a serum composition due to differences in diet and food culture, and internal factors such as hormonal differences between genders, the stage of menstruation, the age of patients, and the copresence of other diseases, infections, and inflammation in a patient. Narrowing the molecular window establishes a foundation to minimize numerous possible influences that can deteriorate the accuracy of cancer diagnosis. Further study focused to these factors will be required in future to verify the degree of the individual influences.

## Supporting Information Available

Additional data, patient information, and adopted experimental and mathematical methods (PDF)
