# Supplementary material for: Improving Vibrational Spectroscopy Prospects in Frontline Clinical Diagnosis: Fourier Transform Infrared on Buccal Mucosa Cancer
Source: Anal Chem. 2022 Sep 26;94(40):13642–6. doi: 10.1021/acs.analchem.2c02496 (PMC9558084; doi:10.1021/acs.analchem.2c02496)
Supplement: Supplementary file 1 — ac2c02496_si_001.pdf [file ac2c02496_si_001.pdf]

Supporting information

Improving Vibrational Spectroscopy Prospects in Frontline Clinical Diagnosis – FTIR on Buccal Mucosa Cancer

Edward Duckworth[a], Arti Hole[b], Atul Deshmukh[d], Pankaj Chaturvedi[c], Murali Krishna Chilakapati**[c,b,e], Benjamin Mora[a], Debdulal Roy*[a]

a. Swansea University, Singleton Park, Swansea, SA28PP, Wales, UK.
b. Advanced Centre for Treatment, Research and Education in Cancer, Kharghar, Navi Mumbai, 410 210, India.
c. Tata Memorial Center, Head and Neck Surgical Oncology, Dr. E Borges Road, Parel, Mumbai 400012, India.
d. Center for Interdisciplinary Research, D. Y. Patil Dental College, Nerul, Navi Mumbai 400706, India.
e. Department of Life Sciences, Homi Bhaba National Institute, Mumbai, India

Authors for correspondence: *deb.roy@swansea.ac.uk and **mchilakapati@actrec.gov.in

Table of Contents

1. Patient information S-2
2. Detailed methodology S-4
3. Sample preparation S-5
4. FTIR Instrument Specifications: S-7
5. Pre-processing of FTIR spectra: S-7
6. Post-processing Method using PCA and SVM S-8
7. Measuring confidence S-12
8. DOME compliance S-13
9. Additional example graphs and tables S-17

* deb.Roy@swansea.ac.uk or d.roy@cantab.net

Abstract:

The following materials contain additional information not present in the main manuscript for additional clarity and confidence in the results and greater replicability of our methods. For replicability, of particular note are sections 3-7, our sample preparation, Instrument specification. For additional confidence, table S6 includes an experiment comparing our Cross validation with a traditional leave-20%-out method. For additional data and graphs that couldn't be included, see section 9.

1. Patient information

Table-S1: patient age distribution

	Average age	Standard Deviation (SD)	2SD error
Healthy (with and without tobacco habit)	37	10.9	5.5
Cancer	59	15.7	6.7
Premalignant	42	11.2	4.8

Table S2: Human serum band assignments

Wavenumbers	Assignments
3280	H–O–H stretching
2957	Asymmetric CH_3 stretching
2920	Asymmetric CH_2 stretching
2872	Symmetric CH_3 stretching
1635	Amide I of proteins νC=O (70– 85%)/νC–N/δN–H
1537	Amide II of proteins δN–H (40– 60%)/νC–N (18–40%)
1453	CH2 scissoring
1396	C=O stretch of COO–
1230–1330	Amide III (N–H bend in plane and C–N stretch)
1311	CH_2 twist
1242	Asymmetric PO^{2-} stretch
1170	Ester C–O asymmetric stretch
1110	ν(CN)/δ(CH)
1079	C–O stretch
1047	C-O stretch
926	C-C-N Backbone, C-C stretch
905-915	$-CH=CH_2$

2. Detailed methodology

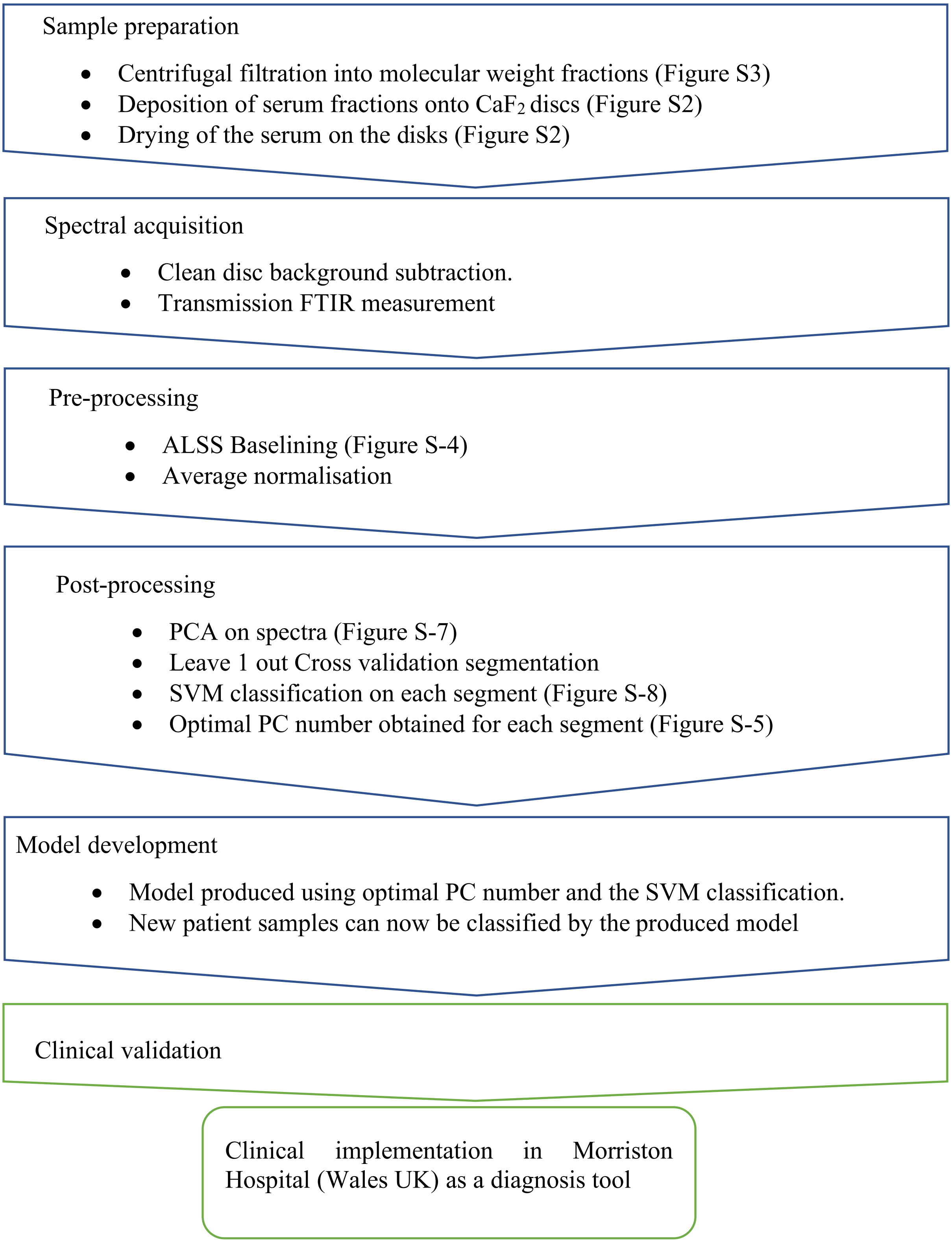

Figure S1: Outline of the process followed for classification of patients from sample collection to clinical implementation in a hospital.

3. Sample preparation

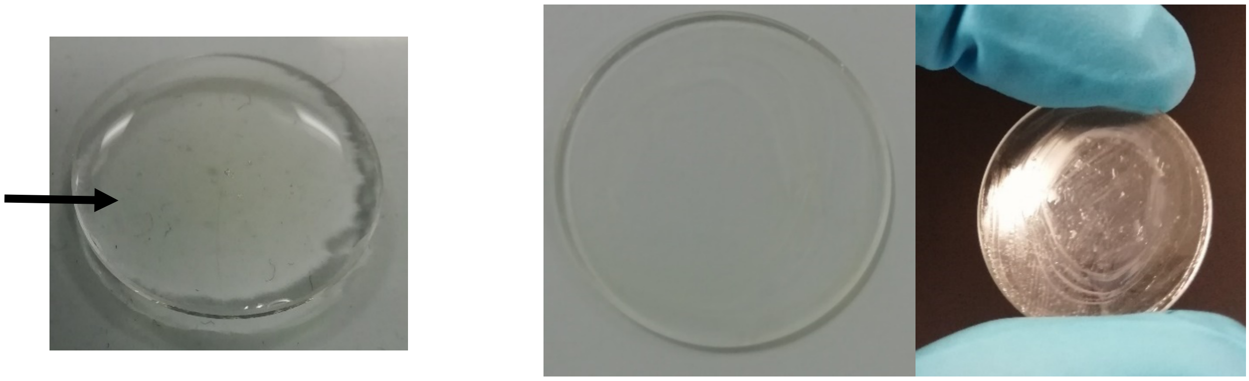

Figure S2: images of the droplet deposition before and after drying. Serum fraction was diluted 24:1 and deposited on the disc, ensuring coverage to the edges. The layer is more even than without these precautions, but there is still evidence of the ‘coffee ring effect’ producing rings of higher weight components at the edge of the drying zone.

See figure S1 for a complete flowchart of the process. You can see an example of the drying process used in figure S2. 1:24 ratio was determined experimentally. It is selected to give sufficient volume to coat the whole disc while keeping the amount of serum material deposited after drying in acceptable absorbance ranges for the FTIR measurement. Potential sources of error are present from the dilution steps not being sufficient to completely eliminate variable deposition, i.e. the ‘coffee ring effect’. In the HMW sample, there was also sometimes evident cracking from the drying process. Although the location of acquisition was controlled, it is possible some key spectral shifts may have been reduced as a result of these uncontrolled deposition patterns. The consistency of the innermost area (Figure S2) made with the obvious choice for the controlled detection location with the lowest average error deviation of 0.45 % absorption, the middle and outer having 0.75 and 8.1 % absorption respectively.

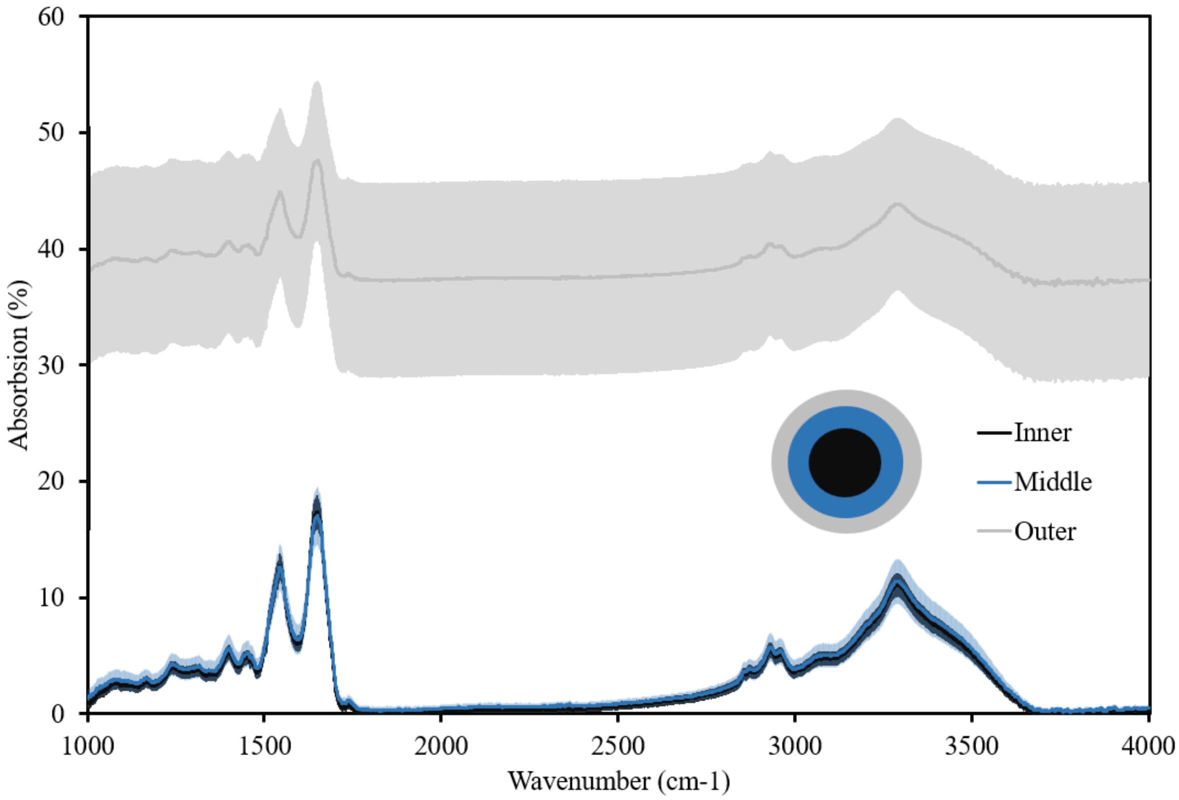

Figure S3: raw absorption spectra averages taken from each disc section by transmission FTIR microscopy. The inner most ~8 mm, the middle ~8-16 mm and the outer ~16-25 mm diameter. One can observe the error increasing as the spectra approach the edge of the disc. The absorption is far higher at the educe due to both the coffee ring effect and contributions of the edge of the disc being within the ~3mm detection aperture.

Centrifugal Filtration for Molecular-weight Windowing

- Place samples in the top compartment of the centrifugal filtration unit
- Centrifuge for recommended time based on kDa rating (10-30 mins) at 14,000 G.
- Dilute sample into 500 µl of ultrapure water before deposition. If >100 kda concentrate use only 10 µl, up to 20 µl for the rest.
- Let samples dry in ambient conditions (~2 hours) before recording IR spectra.

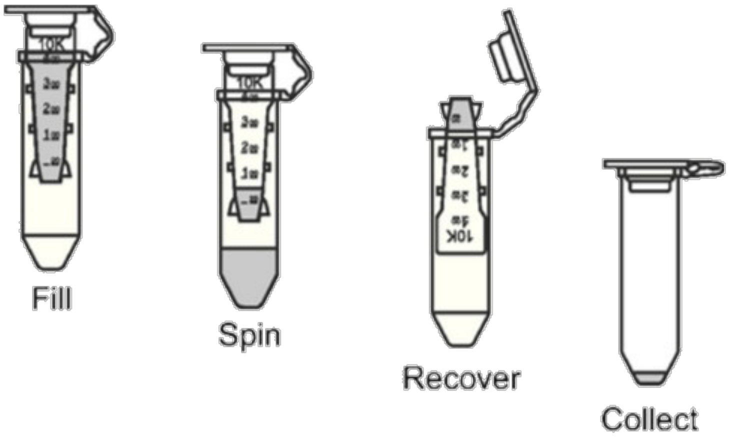

Figure S4: Schematic view of concentrating samples using a Merck-Millipore centrifugal filtration unit

4. FTIR Instrument Specifications:

A Perkin Elmer spectrum 2 FTIR was used for the analysis and the spectral data was acquired using the integrated Perkin Elmer Spectrum software. The resolution was 4cm^{-1}, range 750-4000cm^{-1}, 4 5s acquisitions. Each reading was repeated 3 times for each sample, adjusting the beam location on the disc by 2mm for each repetition. Background of a clean disc was taken beforehand for subtraction. Instrument specifications: 8,300 – 350 cm-1 long-life IR source, Dynascan™ Michelson interferometer with proprietary extended range KBr beam splitter, LiTaO3 detector and a 12mm diameter spot size. The instrument uses an over-sampling delta-sigma converte, and a small amount of zero filing and cubic spline interpolation.

5. Pre-processing of FTIR spectra:

Spectra were pre-processed with a background correction using the asymmetric least squares smoothing (ALSS) method. The method uses a smoothing algorithm with an asymmetric weighting of deviations to get a baseline estimator. This allows a corrective baseline to be quickly obtained while retaining the signal peak information. Baselining

was followed by average normalisation by dividing by the average intensity for each spectra. Spectra were also trimmed to 800-1800 cm^{-1} to focus on the fingerprint region (internal testing produced more efficient classification when doing so) and to remove some obstructive noise between 750-800 cm^{-1}.

6. Post-processing Method using PCA and SVM

It is often observed in chemometric analysis of spectroscopy data that when the markers or biochemical species contributing to a classification are few, commonly, fewer than 5 PCs are adequate to explain most of the variance and therefore a larger number of PCs are considered to add to overfitting, and result in a poor cross-validation accuracy. However, in the case of our FTIR data, the cancer biomarkers contributing to classifications are unclear, potentially have multiple components/peaks and also likely to contribute weaker signals in a spectrum due to very low concentrations. It is our observation that higher PCs can contribute to increased accuracy, provided the overfitting issue can be handled with utmost care. We deal with this by rigorous cross-validation to measure any overfitting and to identify a suitable number of PCs to use for the classification.

The complete leave-one-out cross validation removes all the samples from one patient (3 repeats), produces a model of the remaining patients' data and tests it on the 3 left-out samples. This is repeated until all the patients have been left out of the model once and the % of correct classifications provides the cross-validated sensitivity/specificity/accuracy. This method is repeated for each number of principle components, up to 50, and the accuracies are plotted. With SVM the graph tends to plateau after a certain number of components, mirroring a plot of explained variance (Figure 2). The accuracy after the graph has plateaued, and the corresponding number of principle components, were then chosen to produce the ideal model.

It is important to use statistics to discern the spectra. The eye test can pick out certain trends, especially if presented as difference graphs as in Figure 1 b-e. However, these observable trends can only account for so much of a classification. To reach the higher %, more minor and non-universal spectral differences must be considered. The use of machine learning based classification can discern that which our eyes cannot, finding the key minor peak differences and ratios that bring the classifications from the 70%s to the 80 and 90%s.

Principal component Analysis (PCA) is a statistical procedure that will take a set of spectrograms and use orthogonal transformation to convert them into a set of linearly uncorrelated variables termed ‘principal components’. The process is designed to create the variable with the highest possible variance first, followed by the next highest possible that is orthogonal to the previous component(s) and so on (Figure S7). Typically, one would only look at the first few principal components, provided that they accounted for a high enough percentage of the variance.

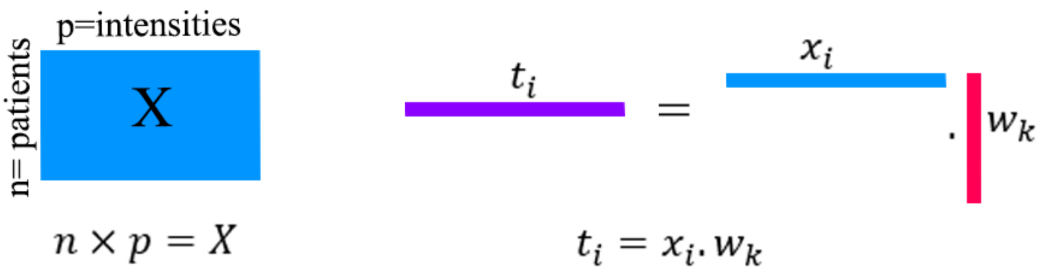

Figure S5: Given our spectral dataset of **n** patient samples and **p** intensities at each wavenumber, we can make an **n**×**p** matrix **X**. Principle component scores **t** can be found for the **i**-th sample from a transformation **w** defined as a set of **p** dimensional vectors. The first of these has maximum variance and therefore is defined $\boldsymbol{arg\,max}\{\|\boldsymbol{Xw}\|^2\}$ where **w** is a unit vector. As the components are orthogonal eigenvectors of **X**, subsequent components can be found by subtracting the previous components from **X** and repeating.

Often the dataset is plotted as a scatter graph on a 2D map of two of these components, typically the first two with the highest variance. This can result in distinct clustering of the data points, with spectrograms exhibiting similarities being placed together. The hope is that these clusters will match the classes - for example, one cluster being serum from diseased patients and the other being healthy samples.

If the clustering is observed, one can then separate the clusters into classes with a line and make the assumption that any new data point that falls on one side of the line will be a member of that class. The measure of how effective a predictor one's method is, is termed accuracy and is a combination of the 'specificity' and 'sensitivity' of the method. Using the example of determining if a sample is positive for a disease, sensitivity is a percentage measure of how many true positives the method identifies, and specificity is a similar measure of the proportion of true negatives. More than 2 components are typically needed for effective classification and thus we need an effective method to draw the optimal separation 'line' for us in a higher (>2) dimensional space.

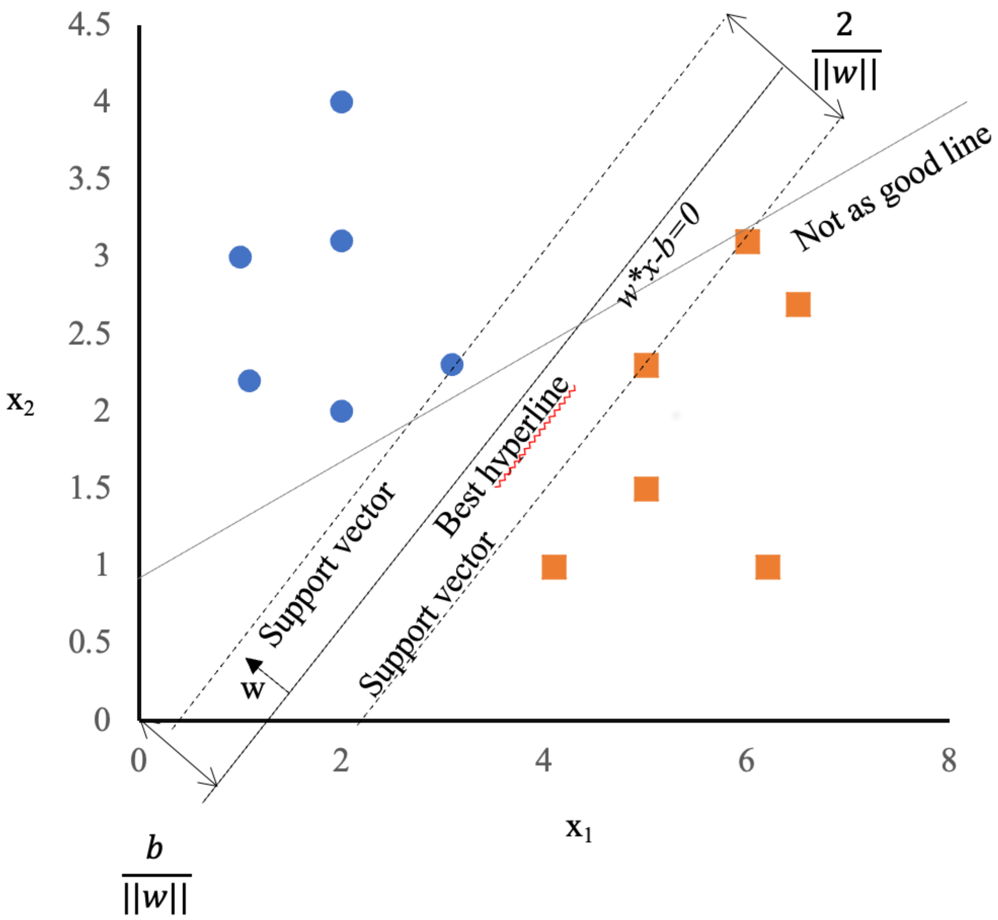

Figure S6: Support vector machine (SVM) is a machine learning algorithm that aims to select the best hyperplane between classes. It chooses this by selecting the hyperplane whose distance to the nearest element of each class is the largest, after prioritising correctly classifying each member. It is also robust to outlier data, by opting to ignore these points through a ‘hinge-loss’ function. Through trial and error, it was discerned that linear SVM was most optimal for the data, though it is classifying in many more than 2 dimensions.

Mathematically, the goal of the SVM function is to minimize:

$$\lambda\|w\|^2 + \left[\frac{1}{n}\sum_{i=1}^{n} \max\left(0, 1 - y_i\left(w^T x_i - b\right)\right)\right]$$

Where **w** is the normal vector to the hyperplane, λ is a tolerance variable and *b* is defined as b/||**w**|| being the offset of the hyperplane from the origin along the normal vector **w**.

This optimisation of the SVM classifier utilises a hinge loss function where the loss (*l*) is given by:

$$l = \begin{cases} 0 \ if \ y.(w.x) \geq 1 \\ 1 - y.(w.x) \ otherwise \end{cases}$$

Meaning anything on the incorrect side of the margin's support vector, not just those incorrectly classified, contributes to the loss.

7. Measuring confidence

Confidence intervals are a suitable method for assessing the confidence in a particular classification method's accuracy. The confidence interval for a Binomial distribution can be given as:

$$Confidence = \mathrm{p} \pm \mathrm{c}\sqrt{\frac{\mathrm{p}(1-\mathrm{p})}{\mathrm{n}}}$$

Where p is the predictive value of the model, e.g. accuracy, used, n is the number of patient spectra and c is a constant based on which confidence interval you are using. These are determined statistically from the standard distribution function. For our purposes, 95% confidence is typically recommended, though there may be situations where either less is sufficient or higher confidence is required – e.g. to avoid potential extreme negative outcomes. For 95%, the constant c is 1.96.

8. DOME compliance

Table S3: Data Optimisation Model Evaluation (DOME) compliance summary[29]

Data	The data size is low for a machine-learning based model. However, this is accounted for by the precision of measurement and overall data quality. A larger scale study would be ideal for further validation.<br>There may be a sight bias between healthy/cancer groups based on age.
Optimization	As relatively quick SVM is used, full leave one out (LOO) cross validation is possible. Each model being produced independently of the left-out patient's test samples.<br>Overfitting is accounted for by using an 'early stop' when the number of PCs used starts to reduce cross-validation accuracy.
Model	SVC used from Python module SKlearn.svm, parameters: linear kernel, tolerance $1x10^{-5}$<br>Output values available in table s1<br>Execution time was typically under 5 mins without excessive optimisation.
Evaluation	Statistical method PCA-LDA was also performed on several of the classifications, and produces similar but typically slightly inferior cross validated results.<br>Performance is consistent.

Spectra were analysed over a range of principle components followed by linear support vector machine (PCA-SVM) classification, and sensitivity and specificity values were obtained by complete leave-one-out cross-validation. All repeats from one sample were left out of each segment and the ability to classify them correctly was assessed. All accuracy values quoted are

these cross-validation results. This was done as, if given enough PCs to use, a model will eventually correctly classify close to 100% of samples. Unfortunately, much of this will be due to overfitting the particular sample set's random noise or random confounding factors (due to an individual's diet etc.). Therefore, using the cross-validation values ensure that we are quoting transferable results that could potentially be used to diagnose a new patient sample. These mathematical functions were implemented in Python using in-built functions from the SciPy and Sklearn modules.

Table-S4: Design of the study and specification of patient numbers and molecular weight regions of serum.

Patients	Study involving whole serum, low mol weight and high mol weight windows of the serum	Study involving the 10-30 kDa molecular weight window
**Buccal mucosa cancers**	42	9
**Premalignant oral conditions (leucoplakia)**	40	9
**Healthy tobacco user**	17	0
**Healthy (not tobacco user)**	27	0
**Total**	126	18

Table-S5: confusion matrix for whole serum, Cancer v Premalignant.

Number of spectra = 240		True clinical diagnosis		
		Cancer	Premalignant	Total:
Model predicted diagnosis	Cancer	True Positive: **113**	False Positive: **18**	131
	Premalignant	False Negative: **13**	True Negative: **96**	109
	Total:	126	114	Accuracy: **87.1%** Confidence : 4%

To further validate this approach, 20% of the data were left out for testing the model and the data from the confusion matrix was compared with the accuracy (See Table-S6).

The results of a validation experiment of the complete LOO methodology to find a suitable number of principle components can be seen in Table S6. Here the PCA-SVM + leave-one-out cross-validation process is used to produce a model on 80% of the patients. The accuracy is slightly lower than the full cohort due to the reduced sample size. The produced cross-validation accuracy is proven to correspond to the accuracy for using the model to classify the 20% left-out data. The 83% accuracy validation is comfortably within confidence of the 86% accurate model.

Table-S6: confusion matrix for whole serum, Cancer v Premalignant, with 20% of data removed for validation. Figures are displayed model/validation.

Model/Validation: Number of spectra = 192/48 (80/20%)		True clinical diagnosis		
		Cancer	Premalignant	Total:
Model predicted diagnosis	Cancer	True Positive: **89/22**	False Positive: **13/6**	102/28
	Premalignant	False Negative: **13/2**	True Negative: **77/18**	90/20
	Total:	102/24	90/24	Accuracy: **86/83%** Confidence : 5/10%

9. Additional example graphs and tables

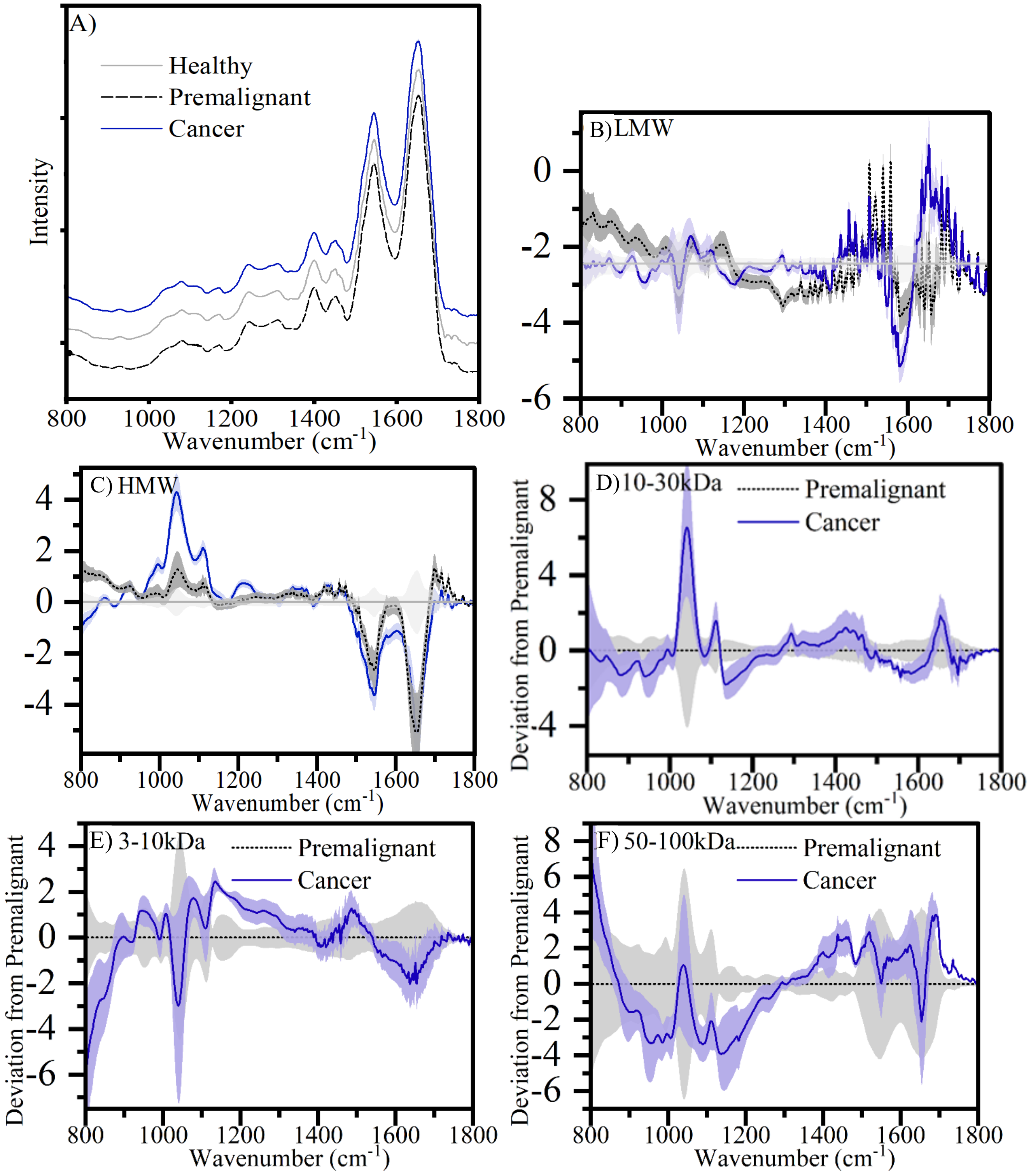

Figure S7: A) Average FTIR Spectra of the healthy, premalignant and cancer patients from whole serum (A-C). Each spectrum is offset for clarity in this graph only. 1σ error is minimal so is indiscernible from the graph at this scale. Difference in the average spectra of cancer patient serum from healthy : B) LMW, C) HMW. Average FTIR Spectra of the cancer patients from premalignant: D) 10-30, E) 3-10 kDa and F) 50-100 kDa windows. Error in faded colour around each line to show level of distinction for each spectrum.

It is obvious from Figure S7(A) that the FTIR spectra acquired from the whole serum are hard to visually discern the cancer patients from the premalignant and healthy patients. To bring out the differences, the healthy patient's spectra are subtracted from the cancer and premalignant spectra the difference spectra are shown in Figure S7(B-F). These plots highlight the clear differences between the sample groups, especially demonstrating how discernible both premalignant and cancer patients are from healthy patients.

Therefore, there are still valuable information to be gleaned in the data from this subset. For example, the observable peak shifts in Figure-1C can be referenced against known chemical signatures[26-28]. An out-of-error shift can be observed in the cancer patient's signal at 900-950, 1537 and 1635 cm^{-1}. The ~900 cm^{-1} shifts could be attributed to alkene carbon double bond bending or C-C bond stretching. The 1557 $cm^{-1}$and 1635 cm^{-1} peaks can be related to the Amide II and I peak respectively (See Table-S-2).

One can also examine the commonalities between the peak patterns for cancer and premalignant samples, the regions with shared peak patterns emphasising the need for the premalignant control so than the cancer specific signals can be discerned, lest those patterns be erroneously incorporated into a spectral biomarker.

Table S7: Additional comparative classifications. Using SVM and LDA alone can result in high classification accuracy but fails in the cross validation, due to overfitting.

Method:		SVM only		LDA only		PCA-SVM	
**Fraction**	**Classifi-cation**	**Original accuracy**	**Cross validated Accuracy**	**Original accuracy**	**Cross validated Accuracy**	**Original accuracy**	**Cross validated Accuracy**
Whole	P v C	80.8	74	100	84.5	100	87.1
	C v All	86.0	78.1	100	84.3	91.6	87.7
	C v H	84.0	62.3	100	79.3	99.0	87.8
LMW	P v C	97.9	77.4	99.5	71.6	99.5	83.4
	C v All	96.2	73.9	99.1	74.3	97.3	75.6
	C v H	99.0	79.8	100	83.7	100	88.0
HMW	P v C	84.8	71.6	100	83.4	93.6	83.0
	C v All	83.6	76.4	100	87.7	95.6	85.9
	C v H	89.3	86.0	99.5	80.7	89.3	89.1
	Average	89.1	**75.5**	99.8	**81.0**	96.2	**85.3**
